# Efficient 1,3-dihydroxyacetone biosynthesis in *Gluconobacter oxydans* using metabolic engineering and a fed-batch strategy

**DOI:** 10.1186/s40643-022-00610-7

**Published:** 2022-11-26

**Authors:** Weizhu Zeng, Xiaoyu Shan, Li Liu, Jingwen Zhou

**Affiliations:** 1 Science Center for Future Foods, Jiangnan University, 1800 Lihu Road, Wuxi, 214122 Jiangsu China; 2 School of Biotechnology and Key Laboratory of Industrial Biotechnology, Ministry of Education, Jiangnan University, 1800 Lihu Road, Wuxi, 214122 Jiangsu China; 3 Engineering Research Center of Ministry of Education On Food Synthetic Biotechnology, Jiangnan University, 1800 Lihu Road, Wuxi, 214122 Jiangsu China; 4 Jiangsu Provisional Research Center of Food Synthetic Biotechnology, Jiangnan University, 1800 Lihu Road, Wuxi, 214122 Jiangsu China

**Keywords:** *Gluconobacter oxydans*, 1,3-Dihydroxyacetone, Metabolic engineering, Dehydrogenase genes, Fed-batch fermentation

## Abstract

1,3-Dihydroxyacetone (DHA) is a commercially important chemical and widely used in cosmetics, pharmaceuticals, and food industries as it prevents excessive water evaporation, and provides anti-ultraviolet radiation protection and antioxidant activity. Currently, the industrial production of DHA is based on a biotechnological synthetic route using *Gluconobacter oxydans*. However, achieving higher production requires more improvements in the synthetic process. In this study, we compared DHA synthesis levels in five industrial wild-type *Gluconobacter* strains, after which the *G. oxydans* WSH-003 strain was selected. Then, 16 dehydrogenase genes, unrelated to DHA synthesis, were individually knocked out, with one strain significantly enhancing DHA production, reaching 89.49 g L^−1^ and 42.27% higher than the wild-type strain. By optimizing the culture media, including seed culture and fermentation media, DHA production was further enhanced. Finally, using an established fed-batch fermentation system, DHA production reached 198.81 g L^−1^ in a 5 L bioreactor, with a glycerol conversion rate of 82.84%.

**Graphical Abstract:**

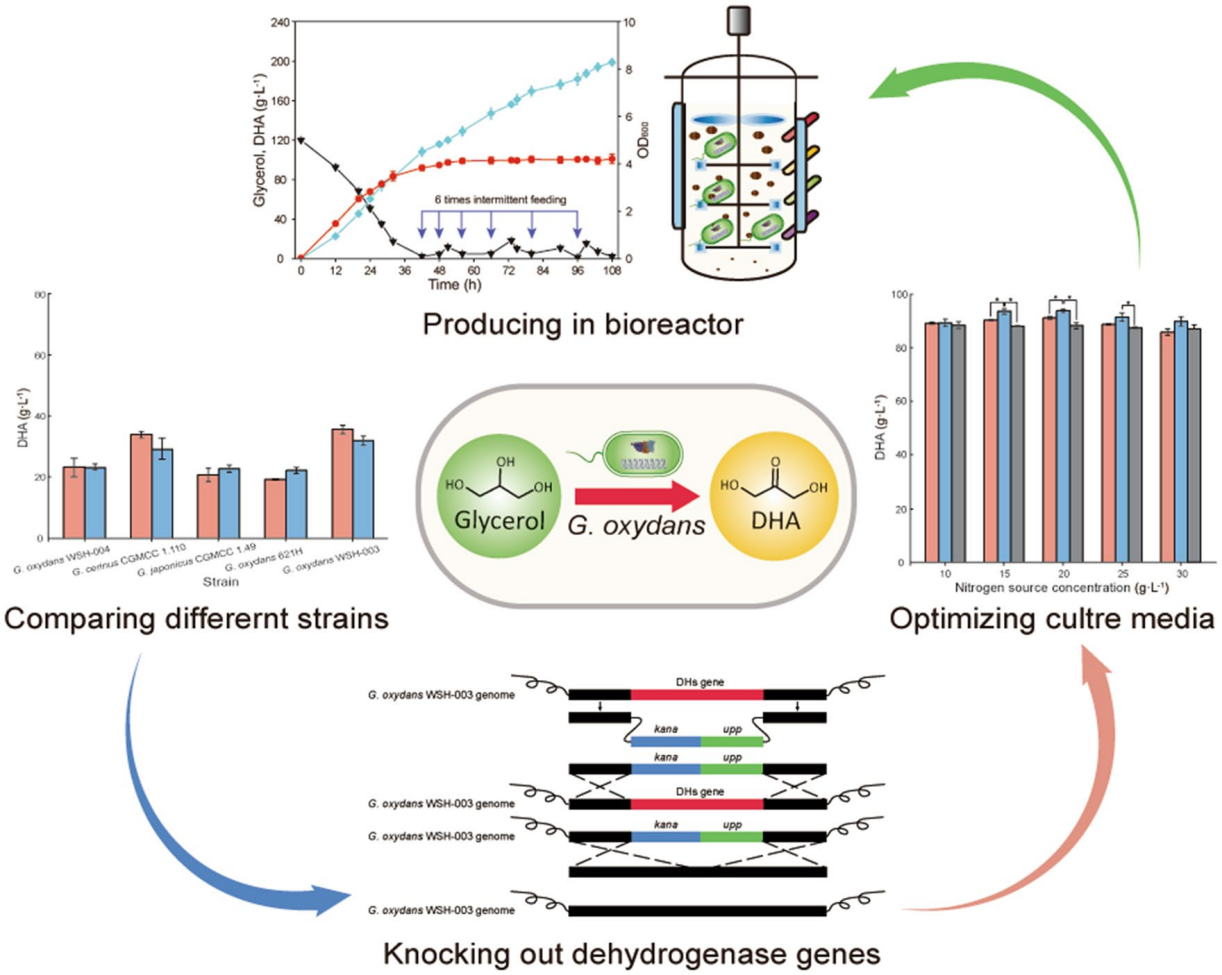

**Supplementary Information:**

The online version contains supplementary material available at 10.1186/s40643-022-00610-7.

## Introduction

1,3-Dihydroxyacetone (DHA) is a commercially important chemical and is widely used in cosmetics, pharmaceutical, and food industries (Dikshit et al. 2017; Turner et al. 2022). DHA reacts with amino acids in the skin cuticle to generate a brown protective film to prevent excessive water evaporation, promote moisturization, and provide sun and anti-ultraviolet radiation protection. DHA does not cause any harmful effects to the skin, as it has been a common active ingredient in sunless tanning skincare preparations (Jankowski et al. 2016; Perer et al. 2020; Perrin, 2020). Due to its ability to turn the skin brown, DHA is also used as a treatment or adjunctive treatment for some skin diseases, such as white spot, psoriasis, and vitiligo (Li et al. 2021b). It was also proposed as a food additive, while DHA effectively promoted body fat reduction and increased endurance (Obeid et al. 2006). In addition, DHA is an essential precursor for the production of different fine chemicals, and serves as a building block for the synthesis of new non-toxic and biodegradable polymers (Dikshit et al. 2018; Korley et al. 2016).

The industrial production of DHA mainly relies on chemical synthesis and biotechnological synthetic routes (Dikshit & Moholkar, 2016a; Tateno et al. 2022). The chemical synthesis method includes formaldehyde condensation and glycerol oxidation; however, the method is problematic as it is difficult to strike a balance between synthesis conditions and material costs. In addition, the method is disadvantageous in terms of low product purity, over oxidation levels, and high costs which limit DHA production (de la Morena et al. 2019). The biotechnological synthetic route is superior in terms of mild reaction conditions, strong specificity, high substrate utilization, a simple process, and therefore, this route has become the main DHA production industrial method (Dobson et al. 2012; Ripoll et al. 2021). Several microorganisms accumulate DHA using glycerol as a substrate, including *Acetobacter*, *Gluconobacter*, *Klebsiella*, and *Pichia*, of which *Gluconobacter oxydans* is generally considered the most desirable and widely used DHA producer (Ko et al. 2020; Lin et al. 2016; Liu et al. 2008).

*G. oxydans* is a classical obligate aerobic bacterium and has a complicated and powerful dehydrogenase system, with an unmatched capacity to effectively and efficiently oxidize alcohols, carbohydrates, and related compounds (Qin et al. 2022). Based on these properties, the organisms is widely used for the industrial production of D-gluconic acid, miglitol, vitamin C, and DHA (da Silva et al. 2022; Li et al. 2022; Ripoll et al. 2021). DHA accumulation commonly relies on a membrane-bound glycerol dehydrogenase (GLDH), which is a pyrroloquinoline quinone dependent enzyme. Studies have reported that GLDH has a wide substrate spectrum as many polyhydric alcohols can be oxidized, such as D-sorbitol, D-arabitol, D-mannitol, and adonitol (Dikshit et al. 2017; Lapenaite et al. 2005). To enhance DHA production, several strategies have been used, including generating high-producing strains using random mutagenesis and gene recombination, optimizing fermentation culture medium and control conditions, and establishing resting cells and immobilized processes (Dikshit et al. 2018; Hu & Zheng, 2011; Kataoka et al. 2021).

In this study, the *G. oxydans* WSH-003 strain, selected from five industrial wild-type *Gluconobacter* strains, generated the best DHA yields. To further enhance DHA production, 16 dehydrogenase genes, unrelated to DHA synthesis, were knocked out by metabolic engineering. We showed that 11 strains, which had 11 different and separate knocked out dehydrogenase genes, significantly promoted DHA production, of which *G. oxydans* WSH-003–4 accumulated a 42.27% higher DHA titer than the original *G. oxydans* WSH-003 strain. After optimizing seed culture and fermentation media compositions, the DHA production was further increased. Finally, using an intermittent fed-batch strategy, the DHA production was enhanced to 198.81 g L^−1^. Our data provide a reference point for enhancing the synthetic efficiency of target compounds in *G. oxydans*.

## Materials and methods

### Microorganisms

Five wild-type *Gluconobacter* strains were used in this study: (1) *G. oxydans* WSH-004, (2) *G. oxydans* WSH-003, (3) *G. oxydans* 621H, (4) *Gluconobacter japonicus* CGMCC 1.49, and (5) *Gluconobacter cerinus* CGMCC 1.110 (Liu et al. 2021). *Escherichia coli* JM109 was used for plasmid construction. A list of study strains is shown in Table 1.

**Table 1:** Study strains and plasmids

Strains and plasmids	Descriptions	Source
*G. oxydans* WSH-004	Wild-type	Preserved in lab
*G. oxydans* WSH-003	Wild-type	Preserved in lab
*G. oxydans* 621H	Wild-type	Preserved in lab
*G. japonicus* CGMCC 1.49	Wild-type	Preserved in lab
*G. cerinus* CGMCC 1.110	Wild-type	Preserved in lab
*E. coli* JM109	Cloning host	Preserved in lab
pMD19-T	Cloning vector	Sangon biotech
pBBR-MCS-2	Host for *kana* gene	Sangon biotech
*G. oxydans* WSH-003–1	Knocked out gene encoding L-idonate 5-dehydrogenase	This study
*G. oxydans* WSH-003–2	Knocked out gene encoding NAD-dependent xylitol dehydrogenase 2	This study
*G. oxydans* WSH-003–3	Knocked out gene encoding alcohol dehydrogenase 3	This study
*G. oxydans* WSH-003–4	Knocked out gene encoding alcohol dehydrogenase 4	This study
G. oxydans WSH-003–5	Knocked out the gene encoding NAD(P)H dehydrogenase (quinone)	This study
*G. oxydans* WSH-003–6	Knocked out gene encoding aldehyde dehydrogenase	This study
*G. oxydans* WSH-003–7	Knocked out gene encoding sorbosone dehydrogenase	This study
*G. oxydans* WSH-003–8	Knocked out gene encoding isocitrate dehydrogenase	This study
*G. oxydans* WSH-003–9	Knocked out gene encoding L-sorbose 1-dehydrogenase	This study
*G. oxydans* WSH-003–10	Knocked out gene encoding NAD(P)H dehydrogenase (quinone) 2	This study
*G. oxydans* WSH-003–11	Knocked out gene encoding zinc-dependent alcohol dehydrogenase	This study
*G. oxydans* WSH-003–12	Knocked out gene encoding gluconate 2-dehydrogenase	This study
*G. oxydans* WSH-003–13	Knocked out gene encoding NADH dehydrogenase (ubiquinone)	This study
*G. oxydans* WSH-003–14	Knocked out gene encoding aldehyde dehydrogenase-like protein	This study
*G. oxydans* WSH-003–15	Knocked out gene encoding glucose dehydrogenase	This study
*G. oxydans* WSH-003–16	Knocked out gene encoding NADH dehydrogenase (quinone)	This study

### Medium and cultivation

The solid slant medium method was used for strain activation and comprised 40 g L^−1^ L-sorbitol, 10 g L^−1^ yeast extract powder, and 20 g L^−1^ agar. Initial seed medium comprised 50 g L^−1^ glucose and 10 g L^−1^ yeast extract powder, and corresponding initial fermentation medium consisted of 50 or 100 g L^−1^ glycerol and 10 g L^−1^ yeast extract powder. Improved seed medium comprised 60 g L^−1^ L-sorbitol and 20 g L^−1^ yeast extract powder, and corresponding improved fermentation medium comprised 50 or 100 g L^−1^ glycerol and 20 g L^−1^ yeast extract powder, 5 g L^−1^ CaCO_3_, 1 g L^−1^ MgSO_4_·7H_2_O, 0.9 g L^−1^ KH_2_PO_4_, and 0.13 g L^−1^ K_2_HPO_4_·3H_2_O (pH 6.2). The final optimized seed medium contained 60 g L^−1^ L-sorbitol and 10 g L^−1^ yeast extract powder, and the final optimized fermentation medium consisted of 100 g L^−1^ glycerol and 15 g L^−1^ yeast extract, 5 g L^−1^ CaCO_3_, 1 g L^−1^ MgSO_4_·7H_2_O, 0.9 g L^−1^ KH_2_PO_4_, and 0.13 g L^−1^ K_2_HPO_4_·3H_2_O (pH 6.2).

DHA strains were incubated on solid slant medium at 30 °C for 48 h. Seed culture and fermentation cultivation were initialized in 500 mL shaking flasks plus 50 mL fermentation seed medium at 30 °C and 200 rpm in a reciprocal shaker (Zhichu, Shanghai, China). Fermentation was performed in a 5 L bioreactor (T&J Bio-engineering, Shanghai, China) containing a 3 L working volume with an agitation speed of 600 rpm, a 2.0 vvm, and 30 °C. The all inoculation size was 6% (v/v).

### Knocking out dehydrogenase genes and constructing engineered strains

Up- and down-stream homologous arms of 16 selected dehydrogenase genes were generated by polymerase chain reaction (PCR) from the *G. oxydans* WSH-003 genome (primer details are provided in Additional file 1). The *kana* gene was amplified from a pBBR-MCS-2 plasmid and individually fused to the up- and down-stream homologous arms of respective dehydrogenase genes. Sixteen fused fragments corresponding to dehydrogenase genes were generated. Then, 16 engineered strains were constructed by integrating the designed fused fragments into the *G. oxydans* WSH-003 parental strain to generate knock-out dehydrogenase gene strains (Liu et al. 2022a; Zeng et al. 2019).

### Analytical methods

Biomass determination: samples at different fermentation timepoints were diluted to an appropriate concentration with 1 M HCl. The optical density was determined at 600 nm.

Glycerol and DHA determination: glycerol and DHA concentrations in fermentation samples were detected using high-performance liquid chromatography (HPLC, Shimadzu, Kyoto, Japan) equipped with an Aminex HPX-87H column (Bio-Rad, CA, USA). HPLC detection conditions: 10 µL of injection volume, 5 mM H_2_SO_4_ of mobile phase with an elution rate of 0.5 mL·min^−1^, and column temperature of 40 °C. DHA was detected using a UV detector at 271 nm and glycerol detected using a refractive index detector.

### Statistical analyses

The experimental data were analyzed by Origin 2019b, and the significant differences were analyzed using student’s *T* tests.

## Results and discussion

### Screening strains for optimal DHA production

**Fig. 1:**
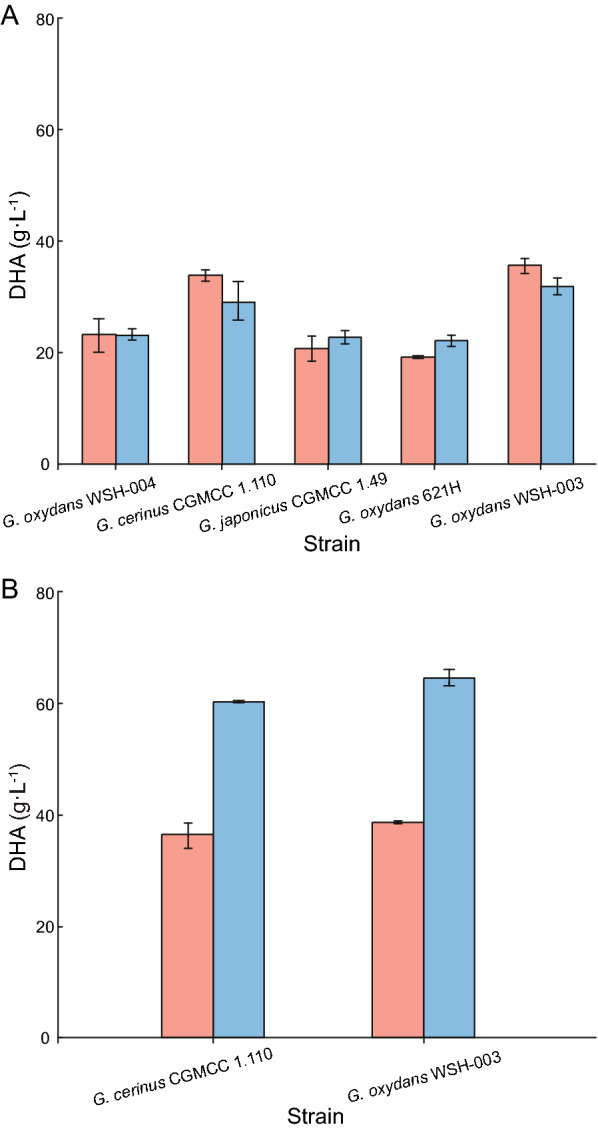
DHA production levels in five wild-type strains. Fermentation was conducted in 500 mL shaking flasks for 72 h. **A** DHA production in five industrial wild-type strains (*G. oxydans* WSH-004, *G. cerinus* CGMCC 1.110, *G. japonicus* CGMCC 1.49, *G. oxydans* 621H, and *G. oxydans* WSH-003) in culture medium containing different glycerol concentrations. **B** DHA production in two wild-type strains in improved culture medium with different glycerol concentrations. Pink; 50 g L^−1^ initial glycerol; Blue; 100 g L^−1^ initial glycerol

For improved DHA production, different *Gluconobacter* sp. strains have been studied and included *Gluconobacter frateurii*, *Gluconobacter thailandicus,* and *G. oxydans* (de la Morena et al. 2020; Jittjang et al. 2020; Poljungreed & Boonyarattanakalin, 2017). To identify optimal DHA production, five wild-type industrial strains, *G. oxydans* WSH-004, *G. oxydans* WSH-003, *G. oxydans* 621H, *G. japonicus* CGMCC 1.49, and *G. cerinus* CGMCC 1.110 were selected and compared (Liu et al. 2021). After well activating, strains were grown in fermentation media containing 50 g L^−1^ and 100 g L^−1^ glycerol as initial substrates. DHA titers in *G. oxydans* WSH-003 and *G. cerinus* CGMCC 1.110 fermentations were higher than the other strains, but when glycerol levels increased to 100 g L^−1^, DHA production decreased (Fig. 1A).

To solve this issue and identify the best DHA producer from *G. oxydans* WSH-003 and *G. cerinus* CGMCC 1.110 strains, both were compared using an improved seed medium that L-sorbitol as carbon source replacing glucose, and the corresponding fermentation medium that adding some significant inorganic salts, such as MgSO_4_·7H_2_O and KH_2_PO_4_ (Dikshit & Moholkar, 2016b; Hu et al. 2010a). As indicated in Fig. 1B, the *G. oxydans* WSH-003 strain performed relatively better, accumulating 64.48 g L^−1^ DHA in 100 g L^−1^ initial glycerol, higher than *G. cerinus* CGMCC 1.110 (60.16 g L^−1^). In addition, in the improved culture medium, DHA accumulation with *G. oxydans* WSH-003 was greatly enhanced at 100 g L^−1^ initial glycerol and was higher than 50 g L^−1^ initial glycerol (38.84 g L^−1^). The conversion rate with 100 g L^−1^ initial glycerol (64.48%) was lower than that of with 50 g L^−1^ initial glycerol (77.68%), but the productivity with 100 g L^−1^ initial glycerol (0.90 g L^−1^ h^−1^) is obvious higher than that of with 50 g L^−1^ initial glycerol (0.54 g L^−1^ h^−1^). Considering the DHA concentration, conversion rate and productivity, addition of 100 g L^−1^ initial glycerol was selected.

### Effects of knocking out dehydrogenase genes on DHA accumulation

*Gluconobacter* has a considerably complex but functional dehydrogenase system, whereby various alcohols/sugars/ketones are rapidly and incompletely oxidized to corresponding sugars/ketones/acids. However, this powerful dehydrogenase system appears to be disordered, such that specific substrates may be oxidized by different dehydrogenases, easily leading to formation of several by-products, which always could be rapidly converted to synthesis of other metabolites or biomass, thereby reducing target product titers (Liu et al. 2022a; Wang et al. 2018). Besides, the competitive dehydrogenases could reduce efficiency of coenzyme for synthesis of target products (Qin et al. 2022; Wang et al. 2016). To enhance the production of specific target compounds in *Gluconobacter*, metabolic engineering strategies involving knock-out or repressing unrelated dehydrogenase genes have been conducted (Liu et al. 2022a; Qin et al. 2021). In previous work, knocking out *mgdh* which encoded a membrane-bound glucose dehydrogenase and *adh* which encoded a membrane-bound alcohol dehydrogenase enhanced DHA titers (Lu et al. 2012). In addition, based on complete genome sequences, more dehydrogenase genes related to alcohols oxidization have been identified in *Gluconobacter* (Prust et al., 2005).

**Fig. 2:**
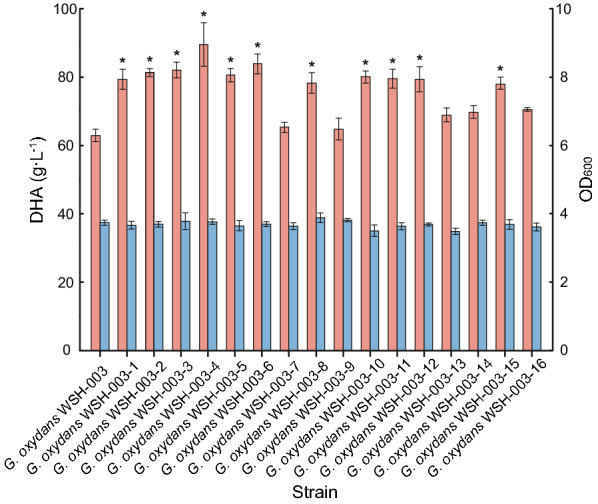
DHA accumulation in knocked out dehydrogenase gene engineered strains. Fermentation was conducted with 100 g L^−1^ initial glycerol in 500 mL shaking flasks for 72 h. When compared with the original wild-type strain (*G. oxydans* WSH-003), 11/16 strains, with knocked out dehydrogenase genes, showed significantly increased DHA production, while the remaining strains showed slight increases. Significant differences in DHA production between engineered and the original wild-type strain were analyzed using student’s *T* tests (*p ≤ 0.05, **p ≤ 0.01). Pink: DHA production; Blue: OD_600_

To investigate the effects of other dehydrogenase knockout strains on DHA accumulation, 16 identified dehydrogenase genes (the identified possible characteristic of these genes were presented in Additional file 1) unrelated to DHA synthesis were selected and individually knocked out. However, according to our experience, these annotated characteristics cannot fully reflect their real functions. In general, they can convert more substrates than the annotated names. The resulting strains were cultured in shaking flasks in a proven fermentation medium plus 100 g L^−1^ glycerol. The wild-type *G. oxydans* WSH-003 strain was used as a control. Results showed that knocking out of these dehydrogenase genes have no obvious effect on cell growth. All engineered strains promoted DHA accumulation; 11 strains induced significant effects, enhancing DHA production by 26.01%, 29.30%, 31.22%, 42.27%, 29.21%, 33.39%, 23.97%, 27.81%, 26.84%, 26.22%, and 23.43%, respectively (Fig. 2). The highest DHA titer (89.49 g L^−1^) was generated by *G. oxydans* WSH-003–4, where the alcohol dehydrogenase 4 was knocked out—productivity was 1.24 g L^−1^ h^−1^. In addition, same to the original strain *G. oxydans* WSH-003, no obvious by-product was detected in fermentation broth with the engineered strains. The specific mechanisms of influences of these dehydrogenases on DHA accumulation need to be further explored in the future. The fermentation characteristics of each engineered strain are shown in Table 2.

**Table 2:** Fermentation characteristics of engineered strains with knocked out dehydrogenase genes

Strains	OD_600_	Production (g·L^−1^)	Conversion (%)	Productivity (g·L^−1^·h^−1^)	Enhancement (%)
*G. oxydans* WSH-003	3.72	62.90	62.90	0.87	/
*G. oxydans* WSH-003–1	3.65	79.26	79.26	1.10	26.01
*G. oxydans* WSH-003–2	3.70	81.33	81.33	1.13	29.30
*G. oxydans* WSH-003–3	3.76	82.54	82.54	1.15	31.22
*G. oxydans* WSH-003–4	3.74	89.49	89.49	1.24	42.27
*G. oxydans* WSH-003–5	3.63	81.27	81.27	1.13	29.21
*G. oxydans* WSH-003–6	3.68	83.90	83.90	1.17	33.39
*G. oxydans* WSH-003–7	3.62	65.36	65.36	0.91	3.91
*G. oxydans* WSH-003–8	3.85	77.98	77.98	1.08	23.97
*G. oxydans* WSH-003–9	3.80	64.58	64.58	0.90	2.67
*G. oxydans* WSH-003–10	3.48	80.39	80.39	1.12	27.81
*G. oxydans* WSH-003–11	3.61	79.78	79.78	1.11	26.84
*G. oxydans* WSH-003–12	3.64	79.39	79.39	1.10	26.22
*G. oxydans* WSH-003–13	3.44	66.96	66.96	0.93	6.45
*G. oxydans* WSH-003–14	3.71	68.82	68.82	0.96	9.41
*G. oxydans* WSH-003–15	3.64	77.64	77.64	1.08	23.43
*G. oxydans* WSH-003–16	3.60	69.18	69.18	0.96	9.98

^*^The OD_600_ and DHA production were the mean value of triple parallel experiments

### Optimizing culture media for DHA production

**Fig. 3:**
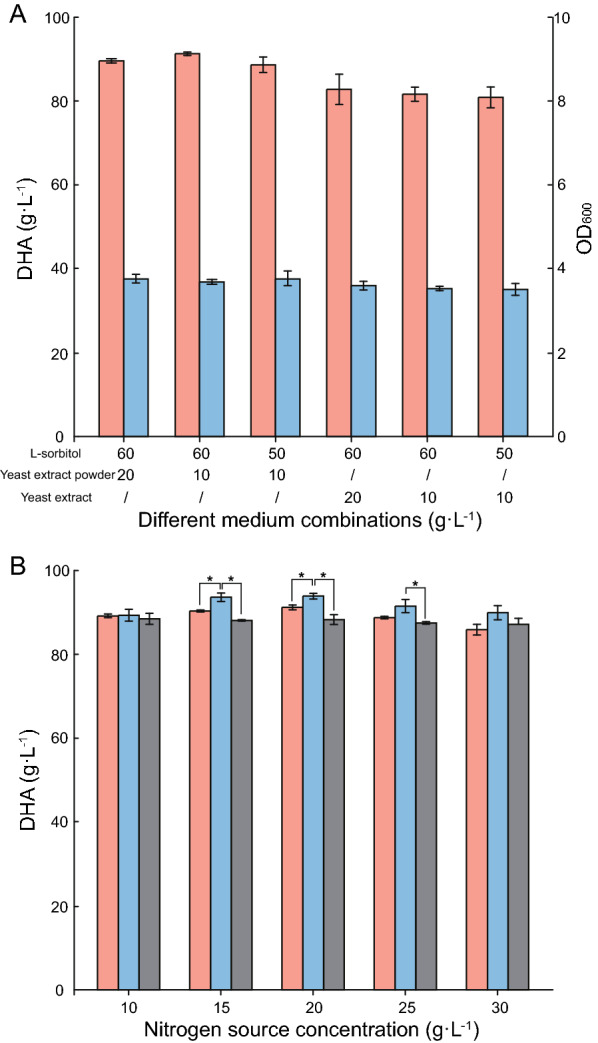
Effects of culture medium on DHA production. Fermentation of engineered strain *G. oxydans* WSH-003–4 was conducted with 100 g L^−1^ initial glycerol in 500 mL shaking flasks for 72 h. **A** Optimization of seed culture medium. Pink; DHA production; Blue; OD_600_. **B** Comparing different nitrogen sources in fermentation medium. Significant differences in DHA production between the yeast extract and other two nitrogen sources (yeast extract powder and corn steep liquor) were analyzed using student’s *T* tests (*p ≤ 0.05, **p ≤ 0.01). Pink; yeast extract powder, Blue; yeast extract, Gray; corn steep liquor

To optimize DHA production with *G. oxydans* WSH-003–4, we investigated the effects of reducing L-sorbitol and yeast extract powder, and replacing yeast extract powder with a cheaper yeast extract in seed culture medium. Appropriately decreased yeast extract powder levels of seed culture medium were more conducive to DHA synthesis, and a high 91.28 g L^−1^ DHA titer was harvested using a combination of 60 g L^−1^ L-sorbitol and 10 g L^−1^ yeast extract powder. However, further reducing the L-sorbitol concentration and replacing yeast extract powder with yeast extract decreased the DHA titer (Fig. 3A). After seed culture medium optimization, the addition of two cheaper nitrogen sources (yeast extract and corn steep liquor) to fermentation medium, and their effects on DHA production, were compared with original yeast extract powder. A relatively higher DHA titer was determined with yeast extract as the nitrogen source; 93.88 g L^−1^ and 93.94 g L^−1^ DHA were generated by 15 g L^−1^ and 20 g L^−1^, respectively. Finally, 15 g·L^−1^ yeast extract addition was selected after comprehensive comparison (Fig. 3B). Comparing the additions in seed culture medium and fermentation medium, it was indicated that a suitable type and concentration of nitrogen source was of significance, since nitrogen sources could affect growth rate of strain and further affect activity of strain.

To bacterially biosynthesize specific target products in a fermentation procedure, culture media, including seed culture and fermentation media are important factors (de la Morena et al. 2019; Wang et al. 2014). A suitable culture medium could improve the target product accumulation. For example, by optimizing seed culture medium composition, ansamitocin production by *Actinosynnema* was increased by 26.8% (Li et al. 2021a). By optimizing different inorganic salts and nitrogen sources in fermentation culture medium, DHA production by *G. oxydans* was promoted and the production period decreased (Dikshit & Moholkar, 2016b; Hu et al. 2010a). In our study, by optimizing seed culture medium composition with respect to L-sorbitol and yeast extract, and comparing different nitrogen sources in the fermentation medium, the DHA production was further enhanced.

### Batch fermentation with different initial glycerol concentrations in a 5 L bioreactor

**Fig. 4:**
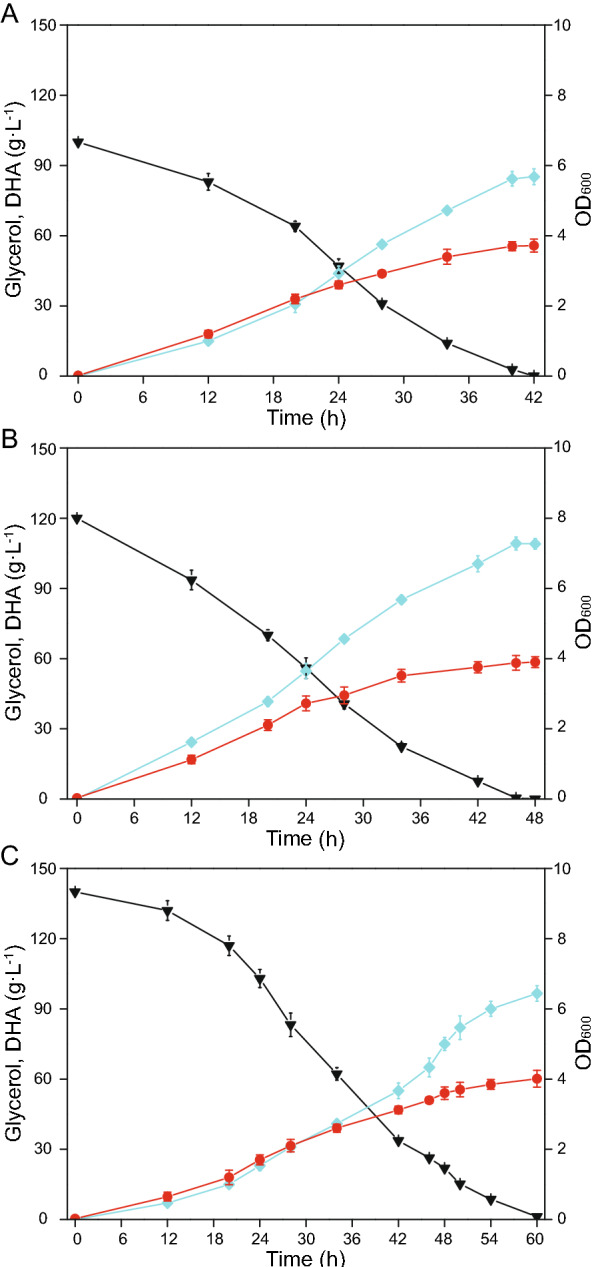
Time courses showing DHA accumulation using different initial glycerol concentrations. Fermentation of engineered strain *G. oxydans* WSH-003–4 was conducted in 5 L bioreactor. **A** Initial glycerol concentration = 100 g L^−1^. **B** Initial glycerol concentration = 120 g L^−1^. **C** Initial glycerol concentration = 140 g L^−1^. Black triangles; glycerol, blue squares; DHA, red circles; OD_600_

With improved seed and fermentation medium, the issue that DHA production decreased when glycerol concentration increased was well-solved, and DHA production further enhanced by optimizing the culture medium in shaking flasks. The effects of different initial glycerol concentrations (100 g L^−1^, 120 g L^−1^, and 140 g L^−1^) on DHA production by *G. oxydans* WSH-003–4 were further investigated in a 5 L bioreactor (Fig. 4). When compared with fermentation in shaking flasks, fermentation period in the bioreactor were significantly shortened. The highest DHA production level was identified at 120 g L^−1^ glycerol for 46 h, reaching 109.18 g L^−1^, with a conversion rate of 90.98% and a productivity of 2.37 g L^−1^ h^−1^, and better than 100 g L^−1^ initial glycerol (its highest DHA titer was 85.25 g L^−1^ for 42 h, with a conversion rate of 85.25% and productivity of 2.03 g L^−1^ h^−1^). When initial glycerol was increased to 140 g L^−1^, the highest DHA titer was just 96.56 g L^−1^, while the fermentation period was 60 h.

Glycerol is a renewable, sustainable, and carbon neutral resource, which is a by-product generated during biodiesel manufacturing processes (Liu et al. 2022b; Luo et al. 2016). It is directly used as a sole carbon source by microorganisms for cell growth and target product synthesis via oxidation and reduction pathways. As it is abundant, available, and easy to absorb, glycerol has become a principal industrial substrate for the production of diverse value-added compounds via biotechnological routes, including the production of erythritol, 1,3-propanediol, organic acids, polyhydroxyalkanoates, exopolysaccharide, biosurfactants, and DHA (Ripoll et al. 2021; Westbrook et al. 2019; Zhang et al. 2021). For DHA production in *G. oxydans*, it was previously shown that high glycerol concentrations inhibited DHA synthesis (Hu et al. 2010b). Similarly, we demonstrated that DHA production efficiency was reduced at 140 g L^−1^ initial glycerol. However, DHA inhibition could be relieved by adding low glycerol concentrations, but titers were also reduced. To ensure production efficiency, an appropriate initial glycerol concentration was necessary (Stasiak-Rozanska et al. 2014).

### Enhanced DHA production using a fed-batch fermentation strategy

**Fig. 5:**
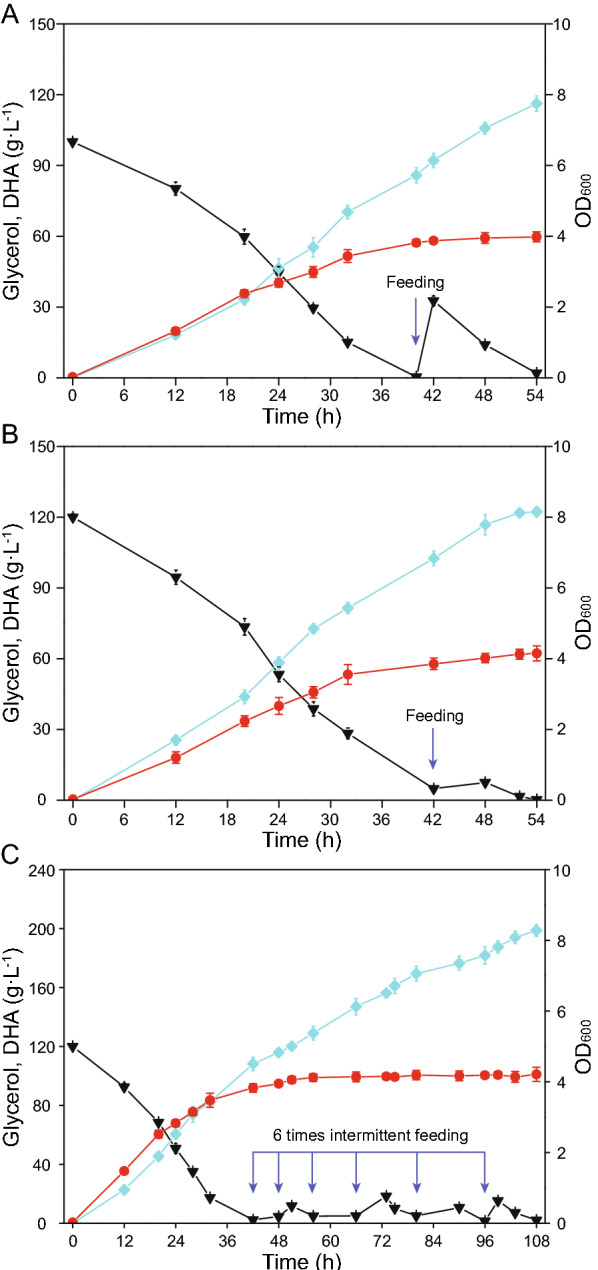
Time courses showing DHA accumulation in fed-batch fermentations. Fermentation of engineered strain G. oxydans WSH-003–4 was conducted in 5 L bioreactor. **A** Initial glycerol addition was 100 g L^ − 1^, and then 40 g L^ − 1^ glycerol was fed at 40 h (Total glycerol addition was 140 g L^ − 1^). **B** Initial glycerol addition was 120 g L^ − 1^, and then 20 g L^ − 1^ glycerol was fed at 42 h (Total glycerol addition was 140 g L^ − 1^). **C** Initial glycerol addition was 120 g L^ − 1^, and then glycerol was intermittently fed (20 g L^ − 1^ each time) at 42 h, 48 h, 56 h, 66 h, 80 h, and 96 h (Total glycerol addition was 240 g L^ − 1^). Black triangles; glycerol, blue squares; DHA, red circles; OD600

Based on batch fermentation data, fed-batch strategy was investigated to improve DHA production by *G. oxydans* WSH-003–4. Using an initial 120 g L^−1^ glycerol, 20 g L^−1^ was fed at 1 time when residual glycerol concentration was < 5 g L^−1^. The process, using an initial 100 g L^−1^ glycerol and feeding 40 g L^−1^ at 1 time, was as a control. The process that using an initial 120 g L^−1^ glycerol and feeding 20 g L^−1^ yielded a better results. 122.23 g L^−1^ DHA was obtained (Fig. 5B), while the control process produced 116.16 g L^−1^ DHA (Fig. 5A). To further enhance the production, an intermittent fed-batch fermentation strategy was proposed, that is using an initial 120 g L^−1^ glycerol, 20 g L^−1^ was fed once residual glycerol concentration was < 5 g L^−1^, and 6 times of feeding was conducted. 198.81 g L^−1^ DHA was generated (conversion rate = 82.84%, productivity = 1.84 g L^−1^ h^−1^) (Fig. 5C). Characteristics of different fermentation processes, based on 120 g L^−1^ initial glycerol, are shown in Table 3.

**Table 3:** Characteristics of different fermentation processes based on 120 g·L^−1^ initial glycerol

Fermentation Characteristics	Batch fermentation	One-dose fed-batch fermentation	Intermittent fed-batch fermentation
Time (h)	46	54	108
Glycerol (g L^−1^)	120	140	240
OD_600_	3.88	4.12	4.23
DHA production (g L^−1^)	109.18	122.23	198.81
Conversion rate (%)	90.98	87.31	82.84
Productivity (g L^−1^ h^−1^)	2.37	2.26	1.84

Fed-batch fermentation processes could enhance target product accumulation. This was particularly relevant to DHA as high glycerol concentrations inhibited DHA synthesis. Based on different producer and corresponding synthetic compound characteristics, diverse fed-batch fermentation strategies have been successfully developed and include a single-dose fed-batch fermentation for 2-phenylethanol production using *Wickerhamomyces anomalus* (Tian et al. 2020), a constant-rate feeding fermentation method for α-ketoglutaric acid production using *Yarrowia lipolytica* (Zeng et al. 2017), and an intermittent fed-batch fermentation method for 2-keto-D-gluconic acid using *Gluconobacter japonicas* (Zeng et al. 2019). In our study, higher DHA production levels were generated, but glycerol conversion rate and productivity were required to improve when compared with reported results (Hu et al. 2010a). In future studies, increased efforts must be made to combine fed-batch fermentation with dissolved oxygen or pH control measures. These two parameters are important factors for alcohol oxidation using *Gluconobacter* (Hanke et al. 2012; Poljungreed and Boonyarattanakalin, 2017).

## Conclusions

To enhance DHA production, the *G. oxydans* WSH-003 strain was selected from five industrial wild-type strains. By knocking out individual dehydrogenase genes unrelated to DHA synthesis, a suite of engineered strains was constructed. The *G. oxydans* WSH-003–4 strain yielded 89.49 g L^−1^ DHA, enhancing production by 42.27%. By optimizing culture media in flasks, DHA production was further enhanced, while the addition of organic nitrogen source in culture media was decreased. Furthermore, using an intermittent fed-batch fermentation strategy, 198.81 g L^−1^ DHA was generated in a 5 L bioreactor, with a glycerol conversion rate of 82.84%. We provide a reference point for the industrial production of DHA and other chemicals using *Gluconobacter*.

### Supplementary Information

**Additional file 1**: **Table S1**. Primers used in this study. **Table S2**. Characteristic of 16 knocked out dehydrogenases.

## Data Availability

All data generated or analyzed during this study are included in this published article and its Additional files.
