# Supplementary material for: Efficient 1,3-dihydroxyacetone biosynthesis in Gluconobacter oxydans using metabolic engineering and a fed-batch strategy
Source: Bioresour Bioprocess. 2022 Nov 26;9(1):121. doi: 10.1186/s40643-022-00610-7 (PMC10992570; doi:10.1186/s40643-022-00610-7)
Supplement: Supplementary file 1 — Additional file 1: Table S1. Primers used in this study. Table S2. Characteristic of 16 knocked out dehydrogenases. [file 40643_2022_610_MOESM1_ESM.docx]

# Efficient 1,3-dihydroxyacetone biosynthesis in *Gluconobacter oxydans* using metabolic engineering and a fed-batch strategy

Weizhu Zeng^1,2,3,4^, Xiaoyu Shan^1,2,3^, Li Liu^1,2,4^, Jingwen Zhou^1,2,3,4*^

^1^ Science Center for Future Foods, Jiangnan University, 1800 Lihu Road, Wuxi, Jiangsu 214122, China;

^2^ School of Biotechnology and Key Laboratory of Industrial Biotechnology, Ministry of Education, Jiangnan University, 1800 Lihu Road, Wuxi, Jiangsu 214122, China;

^3^ Engineering Research Center of Ministry of Education on Food Synthetic Biotechnology, Jiangnan University, 1800 Lihu Road, Wuxi, Jiangsu 214122, China;

^4^ Jiangsu Provisional Research Center of Food Synthetic Biotechnology, Jiangnan University, 1800 Lihu Road, Wuxi, Jiangsu 214122, China.

* Corresponding author: Jingwen Zhou

Mailing address: Science Center for Future Foods, Jiangnan University, 1800 Lihu Road, Wuxi, Jiangsu 214122, China

Phone: +86-510-85918310, Fax: +86-510-85918310

E-mail: zhoujw1982@jiangnan.edu.cn.

## Table S1 Primers used in this study

| **Primers** | **Sequence (5′–3′)** |
| --- | --- |
| 1-up-F | ATCATCTGCCTTATCGGACAAG |
| 1-up-R | GACTCATTCCAAATACCTCTCTGAACAACATGAGATGAAGAGGC |
| *Kana*-F | GAGGTATTTGGAATGAGTCGCCGTCA |
| *Kana*-R | TTGAAGACGAAGACGATT CCTTTCATAGAAGGCGGCGGT |
| 1-down-F | AATCGTCTTCGTCTTCAAATCCTGGGT |
| 1-down-R | GCGGTTCTGCGAAGCCATTT |
| 2-up-F | GTGGCAAAGTTCATTTCATTGCTTG |
| 2-up-R | GCGACTCATTCCAAATACCTCATTTCACCTTGGTGCGCTCAATGGCG |
| *Kana*-F | GAGGTATTTGGAATGAGTCGCCGTCA |
| *Kana*-R | TGATTGCACCACCGTGCTCTCCTTTCATAGAAGGCGGCGGTGGA |
| 2-down-F | AGAGCACGGTGGTGCAATCAAGCAT |
| 2-down-R | TGCAGATCACGCGCGTTACG |
| 3-up-F | CTCTCATGGCTGATACAATGCTGGC |
| 3-up-R | GACTCATTCCAAATACCTCGGTATGTGCTTTCTAAAGTCTCAGGACC |
| *Kana*-F | GAGGTATTTGGAATGAGTCGCCGTCA |
| *Kana*-R | TTACGTATGTATGTCGAGG CCTTTCATAGAAGGCGGCGGT |
| 3-down-F | CCTCGACATACATACGTAATACAGACGG |
| 3-down-R | TAGCAACGTCAAGGCGATTATCTGAA |
| 4-up-F | GGCCAGAAAATTCGATACGGACGA |
| 4-up-R | GACTCATTCCAAATACCTCGGAATTTCCTTTTCTGGGAAGACGGAT |
| *Kana*-F | GAGGTATTTGGAATGAGTCGCCGTCA |
| *Kana*-R | CATGATCAGTCTTCTTTACG CCTTTCATAGAAGGCGGCGGT |
| 4-down-F | CGTAAAGAAGACTGATCATGTGGTGAAAA |
| 4-down-R | ACTCCATCAGCAACGCTTTCACG |
| 5-up-F | GGGTATTTACCGATGTCAGGCCAGAT |
| 5-up-R | GACTCATTCCAAATACCTC TCACGCACTTTCAGGTAACCACGA |
| *Kana*-F | GAGGTATTTGGAATGAGTCGCCGTCA |
| *Kana*-R | CATAAATCCTGGTTCATCC CCTTTCATAGAAGGCGGCGGT |
| 5-down-F | GGATGAACCAGGATTTATGTACTGGCG |
| 5-down-R | GGTATGACCGCCAGCAATCAGG |
| 6-up-F | GAGATGTTCCATGAATCTGGGGATAAGAG |
| 6-up-R | GACTCATTCCAAATACCTC TGACTGCTCCTGTATTCAGGGCG |
| *Kana*-F | GAGGTATTTGGAATGAGTCGCCGTCA |
| *Kana*-R | GACTGCCTTCAGAGAAA CCTTTCATAGAAGGCGGCGGT |
| 6-down-F | TTTCTCTGAAGGCAGTCTGGGAAAC |
| 6-down-R | GGATAACTCCAGTTCGACGAGCCA |
| 7-up-F | GCGTTCTGCCTGAGCTGT |
| 7-up-R | GACTCATTCCAAATACCTC CGCCGTTTCAGTTTCTGCT |
| *Kana*-F | GAGGTATTTGGAATGAGTCGCCGTCA |
| *Kana*-R | CGTAATCGAAACCAGTCATACCTTTCATAGAAGGCGGCGGT |
| 7-down-F | TATGACTGGTTTCGATTACGTTGTCGTTGGTGGCGGCT |
| 7-down-R | AATGGAACTGAAGATCCGGGCA |
| 8-up-F | CCTGATGAATAAAGTACAGCTTGGCGC |
| 8-up-R | GACTCATTCCAAATACCTC GGCGTATCCTGTCTGTCCATGCT |
| *Kana*-F | GAGGTATTTGGAATGAGTCGCCGTCA |
| *Kana*-R | TTCATGATGTCTGTATCCC CCTTTCATAGAAGGCGGCGGT |
| 8-down-F | GGGATACAGACATCATGAAACAGGTTGG |
| 8-down-R | TAGGCTTCGACAAGCGCAATCCG |
| 9-up-F | TTGGCCTCATTACGCCCTGGAACTT |
| 9-up-R | TTCATGATGTCTGTATCCC ATCAGGAGATCCAGTGCGTCCGTTT |
| *Kana*-F | GAGGTATTTGGAATGAGTCGCCGTCA |
| *Kana*-R | AAGCGCCTGAAAACATTGTCTCCTTTCATAGAAGGCGGCGGTGGAA |
| 9-down-F | AGACAATGTTTTCAGGCGCTTCCTCG |
| 9-down-R | CCAGCCGACCGTCTTCAATGTGCATCT |
| 10-up-F | TCTTGATATTAATGCCGCCATGTTCTCC |
| 10-up-R | GACTCATTCCAAATACCTC CGTTTTCAGCCTGCAATGACATTCG |
| *Kana*-F | GAGGTATTTGGAATGAGTCGCCGTCA |
| *Kana*-R | ATCCAGATCAAAGAAACGA CCTTTCATAGAAGGCGGCGGT |
| 10-down-F | TCGTTTCTTTGATCTGGATGCTTTAGGG |
| 10-down-R | GGCAGCCATGTCACAAAAACCC |
| 11-up-F | AGCTGAAATATAGCAAATCCAACAACGACT |
| 11-up-R | GACTCATTCCAAATACCTC GATCCGACTGTCCTTTTTGCAAAGACTG |
| *Kana*-F | GAGGTATTTGGAATGAGTCGCCGTCA |
| *Kana*-R | TTCATCTCAGATACCAGTCCCTTTCATAGAAGGCGGCGGT |
| 11-down-F | GACTGGTATCTGAGATGAAACAGGGATGG |
| 11-down-R | GGGTTGCCAGCCAGCGG |
| 12-up-F | ACACGACAGATAAGATCAGGAGACTGG |
| 12-up-R | GACTCATTCCAAATACCTC AGTGGTCTCCAGAACGAGGGTG |
| *Kana*-F | GAGGTATTTGGAATGAGTCGCCGTCA |
| *Kana*-R | ATGTTTTTTGGTCATGATCT CCTTTCATAGAAGGCGGCGGT |
| 12-down-F | AGATCATGACCAAAAAACATGCAGATGCC |
| 12-down-R | ATTGCTGACGGGATCATACGGTTTT |
| 13-up-F | AGTCCTGGCATACTTATATTCCTTATACTGAT |
| 13-up-R | GACTCATTCCAAATACCTCCAGCCATCACGAAGTCAATGCTGC |
| *Kana*-F | GAGGTATTTGGAATGAGTCGCCGTCA |
| *Kana*-R | AGGGTTTTCAATGCCTTA CCTTTCATAGAAGGCGGCGGT |
| 13-down-F | TAAGGCATTGAAAACCCTTACGCCG |
| 13-down-R | CACCCATGGCAGCCCGTC |
| 14-up-F | GTTTCACACTTGGATTGATTGGCGGG |
| 14-up-R | GACTCATTCCAAATACCTC TCTCTCCCCCGTGCTTCAATATCA |
| *Kana*-F | GAGGTATTTGGAATGAGTCGCCGTCA |
| *Kana*-R | ATCACGTTTCTGTTTTAACA CCTTTCATAGAAGGCGGCGGT |
| 14-down-F | TGTTAAAACAGAAACGTGATGGATGTGTGT |
| 14-down-R | CCTGCATCGCATATCCGGAAGCATT |
| 15-up-F | ACCTAATGTGATGATGGTTTCGTTCGG |
| 15-up-R | GACTCATTCCAAATACCTCGACACCTCTGTTCTTCAAAAAATGGAAACC |
| *Kana*-F | GAGGTATTTGGAATGAGTCGCCGTCA |
| *Kana*-R | CATGACATTTATTTCCGCT CCTTTCATAGAAGGCGGCGGT |
| 15-down-F | AGCGGAAATAAATGTCATGGTTCGCAC |
| 15-down-R | CGGTTTTCTTCAAGAGTGTCGGCT |
| 16-up-F | ACATGAGAACAGATATCGTGCCGCG |
| 16-up-R | GACTCATTCCAAATACCTC GATGCGTTTTTCCCACTGTTTGAAGT |
| *Kana*-F | GAGGTATTTGGAATGAGTCGCCGTCA |
| *Kana*-R | AAATCTGCCGTTTTAAGAG CCTTTCATAGAAGGCGGCGGT |
| 16-down-F | CTCTTAAAACGGCAGATTTTCTCCATTGAT |
| 16-down-R | CGAGCCAGTCATTTGCCAGACAC |

## Table S2 Characteristic of 16 knocked out dehydrogenases

| **Gene list** | **Characteristics** |
| --- | --- |
| 1 | L-idonate 5-dehydrogenase |
| 2 | NAD-dependent xylitol dehydrogenase 2 |
| 3 | Alcohol dehydrogenase 3 |
| 4 | Alcohol dehydrogenase 4 |
| 5 | NAD(P)H dehydrogenase (quinone) |
| 6 | Aldehyde dehydrogenase |
| 7 | Sorbosone dehydrogenase |
| 8 | Isocitrate dehydrogenase |
| 9 | L-sorbose 1-dehydrogenase |
| 10 | NAD(P)H dehydrogenase (quinone) 2 |
| 11 | Zinc-dependent alcohol dehydrogenase |
| 12 | Gluconate 2-dehydrogenase |
| 13 | NADH dehydrogenase (ubiquinone) |
| 14 | Aldehyde dehydrogenase-like protein |
| 15 | Glucose dehydrogenase |
| 16 | NADH dehydrogenase (quinone) |
